# Self-evaluation of ILCORs ten steps to improve around in-hospital cardiac arrests among Swedish hospitals

**DOI:** 10.1016/j.resplu.2024.100672

**Published:** 2024-06-01

**Authors:** Therese Djärv, Ulrika Karlgren, Araz Rawshani

**Affiliations:** a Department of Medicine Solna, Karolinska Institutet, Stockholm, Sweden; b Emergency Department, Karolinska University Hospital, Stockholm, Sweden; c Swedish Resuscitation Council, Sweden; d Department of Molecular and Clinical Medicine, Institute of Medicine, Sahlgrenska Academy, University of Gothenburg, Gothenburg, Sweden

**Keywords:** IHCA, Continuous improvements

## Abstract

**Objectives:**

Recently, ILCOR unveiled the ground-breaking global initiative *“Ten Steps Toward Improving In-Hospital Cardiac Arrest*” (IHCA).

**Aim:**

To generate a baseline of how well the ten steps currently function in Sweden, in order to better target educational interventions.

**Material and methods:**

A survey was created using an online form application (Google Forms) and sent to CPR coordinators and physicians in charge of CPR at all 74 Swedish hospitals participating in the Swedish Registry for Cardiopulmonary Resuscitation (SRCR). Hospitals were asked to self-evaluate their functionality on each step on a ten-point scale ranging from 1 “Not present or not functioning at all” to 10 “Very well-functioning”. Data regarding number of IHCA and their survival during 2018–2022 was gathered from the SRCR.

**Results:**

A total of 34 out of 74 (46%) Swedish hospitals participated in the survey, collectively representing 59% (7,113 out of 12,070) of IHCA cases in SRCR. The responding hospitals were satisfied with the functionality of just over half of the steps currently (median 60%, range 30–90%). The steps with the highest proportion of satisfied hospitals were found for step 6-rapid response systems (85%) and 7-guideline-based resuscitation (94%), while the steps with lowest proportion of satisfied hospitals were found for step 4-goals of treatment (32%) and step 9-person centred culture (18%). About half of participating hospitals expressed intent to prioritise upcoming years’ work on step 1- infrastructure, step 3- effective education and step 5- stop preventable IHCA.

**Conclusion:**

The conclusion is that most hospitals judge themselves to be well-functioning on many of the ten steps, but steps involving effective education might need attention, as well as the tolerance for presence of preventable IHCA **being** low.

## Introduction

Recently, ILCOR unveiled the ground-breaking global initiative *“Ten Steps Toward Improving In-Hospital Cardiac Arrest*” (IHCA).^1^, ^2^ Although in-hospital cardiac arrests (IHCA) receives less research attention compared to OHCA, it still accounts for about one-quarter of all cardiac arrest, underscoring the huge clinical significance.^3^, ^4^ Survival has gradually increased over the last decade.^3^ Yet, the vast majority succumbs, which is unfortunate given that a large portion of IHCA are predictable and/or preventable,^5^, ^6^, ^7^, ^8^ i.e. a deterioration could be foreseen and either stopped or determined as expected and death is no longer avoidable. Further, the first three of the four links in the chain of survival are completed within 3 min for more than three-quarters of the IHCA cases,^3^, ^9^ and it is therefore possible that improvements might need to be based on a broader concept.^10^ Therefore, a comprehensive survey was conducted with the overall aim to generate a baseline of how well the ten steps currently function in Sweden. The baseline will be used to better target educational interventions on a national level as well as to encourage hospitals to learn about the ten steps. Follow-up questionnaires will be sent after three and five years, respectively, covering actions taken by the hospitals and results will be aligned to number of IHCA and survival in Swedish Registry for Cardiopulmonary Resuscitation (SRCR).

## Method

A survey was created using an online form application (Google Forms) and sent to CPR coordinators and physicians in charge of CPR training at all 74 Swedish hospitals participating in the SRCR.^11^ Information about the survey was given during a national registry meeting on the 23rd of November 2023 and thereafter sent per email with a link to the original publication of the ten steps (Supplementary Table A). Up to three reminders were sent. The questionnaire was open for response from the 30th November 2023 to the 4th of March 2024. Hospitals were asked to self-evaluate their functionality on each step on a ten-point scale ranging from 1 “Not present or not functioning at all” to 10 “Very well-functioning”. Answers graded 1 to 4 were classified as “Unsatisfactory” (red in Table 1) and answers graded 7 to 10 were classified as “Satisfactory” (marked in green in Table 1). The percentage of hospitals with satisfactory functionality per step as well as the percentage of satisfying steps per hospital were presented. Data regarding number of IHCA during 2018–2022, proportion of all Swedish IHCA per hospital as well as survival to discharge were gathered from the SRCR. Survival was calculated based on those with a reported outcome. Informed consent was obtained by answering the survey, no further ethical approval was retrieved.

**Table 1:** Self-evaluated level of functionality per ILCORS “Ten steps to improve around IHCA^1^″ among Swedish hospitals.

## Results

A total of 34 out of 74 (46%) Swedish hospitals participated in the survey, collectively representing 59% (7,113 out of 12,070) of IHCA cases in SRCR (Table 1). The overall survival to discharge between 2018 and 2022 was 34%. Missing outcome, i.e. survival, were found in 1601 (13%), with 22% (358) of these occurring within the initial year of the COVID-19 pandemic, commencing from its onset on the March 16, 2020^12^, and extending twelve months thereafter. In total, 33 hospitals had more than the SRCR’s goal for missing outcome, i.e. 5% missing; 23 (70%) of them were non-responders (data not shown). Further, out of the 32 hospitals with at least a 5% lower survival than the overall survival in Sweden, 23 (72%) were non-responders to the survey.

The responding hospitals were satisfied with the functionality of a just above half of the steps currently (median 60%, range 30–90%) (Table 1). No clear pattern was found between patient survival and satisfaction with certain steps or total number of steps. The steps with the highest proportion of satisfied hospitals was found for step 6-rapid response systems (85%) and 7-guideline-based resuscitation (94%) while the steps with lowest portion of satisfied hospitals was found for step 4-goals of treatment (32%) and step 9-person centred culture (18%).

**Fig. 1:**
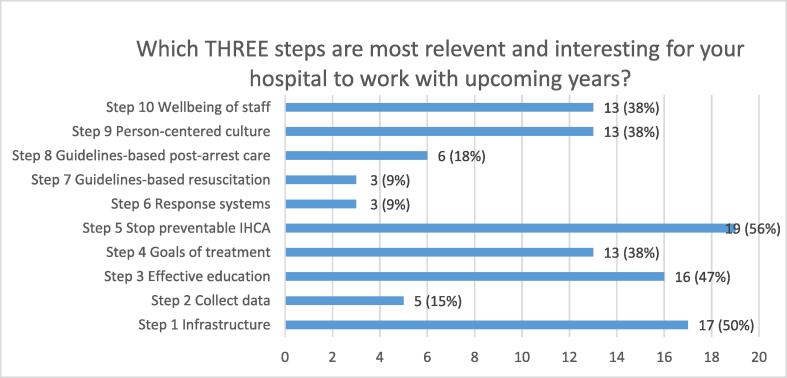
Self-selected steps to work with upcoming years out of ILCORS “Ten steps to improve around IHCA^1^″ among Swedish hospitals.

Regarding the three steps that the hospital would like to improve in the coming years, about half of the participating hospitals expressed intent to prioritize step 1- infrastructure, step 3- effective education and step 5- stop preventable IHCA (Fig. 1).

## Discussion

This is the first published self-evaluation of ILCORs ten steps on a national level, offering a pioneering insight into the landscape of IHCA management from hospitalś perspective. These insights will be useful when implementing ILCOR's directives into tangible actions as well as to guide educational interventions in similar settings. Finally, it provides a baseline for future evaluations of the steps aligned to reductions in IHCA incidence and concomitant elevations in survival ratios, epitomising the overarching goal of ILCOR's initiative.

There was clear alignment between steps self-evaluated as not satisfying and the three steps selected as of interest for future focus except for step 5- stop preventable IHCA. While Step 5 elicits considerable satisfaction, its continued relevance underscores the imperative for ongoing refinement. Since 2016 most Swedish hospitals have implemented the national early warning score (NEWS),^13^ but the compliance between the trigger and the clinical response might still be lagging.^14^ The satisfaction with steps focusing on following guidelines, steps 7 and 8, most likely marks a strong culture of following guidelines, especially intra-arrest^9^, ^15^ as well as low interest in working with step 6- rapid response systems, which indicates an implemented mature system even if there is still room for improvments.^16^ Likewise, step 2- collect data, the participating hospitals already work closely with the national registry established in 2007 for IHCA.^11^

We lack information about reasons for hospitals not participating in the survey. However, of note is that the hospitals with both higher rates of missing outcomes than the goal of the registry as well as poorer survival than the overall survival in Sweden were often non-responders, this might indicate a less structured ongoing work or awareness of the functionality of each step within the hospital.

Limitations include the response rate, the fact that the self-assessment is highly subjective and lack of validation of interpretation of each step and calibration of the tenth-grade scale between hospitals. Future studies might need to develop an objective ranking scale, for example 0–10 points per step summing up to a total score of 0–100 for each hospital, but currently no such tool exits. Strengths lie in the holistic approach, drawing insights directly from frontline practitioners immersed in IHCA and CPR management and the use of the national all-encompassing registry for number of IHCA and survival.

The results could likely be used in settings similar to Sweden and the questionnaire might be copied and run in similar or other settings to generate a baseline and thereafter target inventions. Within Sweden we will use the results to target education interventions such as themes for symposiums and congresses as well as to facilitate sharing of good examples between hospitals at meetings. A follow-up questionnaire is planned in three as well as five years tracking interventions undertaken and their impact on IHCA metrics, including incidence rates and survival outcomes.

Conclusions are that most hospital judge themselves as well functioning on many of the ten steps but steps involving effective education might need attention as well as the tolerance for the presence of preventable IHCA being low.

## Data sharing statement

No additional data exists that is suitable for publication.

## CRediT authorship contribution statement

**Therese Djärv:** Writing – review & editing, Writing – original draft, Visualization, Validation, Supervision, Resources, Project administration, Methodology, Investigation, Formal analysis, Data curation, Conceptualization. **Ulrika Karlgren:** Writing – review & editing, Formal analysis, Conceptualization. **Araz Rawshani:** Writing – review & editing, Visualization, Methodology, Formal analysis, Conceptualization.

## Declaration of competing interest

The authors declare that they have no known competing financial interests or personal relationships that could have appeared to influence the work reported in this paper.
