# Supplementary material for: Self-evaluation of ILCORs ten steps to improve around in-hospital cardiac arrests among Swedish hospitals
Source: Resusc Plus. 2024 Jun 1;19:100672. doi: 10.1016/j.resplu.2024.100672 (PMC11169947; doi:10.1016/j.resplu.2024.100672)
Supplement: Supplementary Data 1 [file mmc1.docx]

**Supplementary table A – Summary of ten steps toward improving in-hospital cardiac arrest quality of care and outcomes, adopted from the original publication^1^.**

| **Theme** | **Step** | **Content** |
| --- | --- | --- |
| Plan and prepare | 1 | Build and support governance and infrastructure for a resuscitation program: create a governance structure with administrative leadership to advance resuscitation excellence. Empower local operational champions and ensure a robust quality improvement program |
|  | 2 | Collect data to measure and improve resuscitation processes and outcomes: use data to assess education, training, performance, and outcomes across the continuum of resuscitation care and to devise and evaluate the impact of quality improvement efforts to ensure their sustainability. Consider participating in resuscitation registries to enable benchmarking and contribute to knowledge transfer |
|  | 3 | Implement effective education and training for resuscitation: create and maintain resuscitation education and training programs for resuscitation, including (1) standardized, in-person resuscitation courses; (2) low-dose, high-frequency in-situ training; (3) team-based training and simulations; and (4) debriefing to learn from resuscitation events. |
| Prevent | 4 | Establish patient and family goals of treatment early and reassess often: proactively and collaboratively engage patients and their families in dialogue regarding their preferences for resuscitation in the context of their values, comorbidities, frailty, and family needs. Recognize that there are patients for whom resuscitation is unwanted or unlikely to result in an outcome that is acceptable to the patient. Ideally, goals of treatment conversations would start in the community and be updated on admission and after any change in clinical condition. |
|  | 5 | Stop preventable IHCA: have systems (eg, early warning systems) and teams (eg, medical/rapid response teams) in place to enable early prediction, identification, and response to deteriorating patients. Provide effective communication to ensure a clear plan and safe transition of care. |
| Perform | 6 | Develop and deploy an effective resuscitation response system: make available a hospital-wide resuscitation response system that is easily and rapidly activated. Ensure a high-quality resuscitation team that includes preassigned, experienced, and interdisciplinary health care professionals. |
|  | 7 | Deliver guideline-based resuscitation care: focus on essential components of guideline-based skills for basic life support, advanced life support, and pediatric life support. These include prompt initiation of high-performance CPR and defibrillation and the use of appropriate and cause-directed interventions. Avoid nonrecommended interventions and early termination of resuscitation |
|  | 8 | Deliver guideline-based post–cardiac arrest care: empower multidisciplinary teams to provide guideline-based post–cardiac arrest care, throughout and beyond hospitalization. Use multiple modalities to inform prognostication. Consider organ donation, when appropriate. |
| Principles and culture | 9 | Implement a person-centered culture of excellence in care: ensure there is informed, culturally appropriate communication with patients and family to support them and to understand their goals, values, and preferences. For cardiac arrest survivors, development of postdischarge plans with targeted resources (eg, primary care, rehabilitation) is crucial for recovery and survivorship. For families of nonsurvivors, offer grief and bereavement resources. |
|  | 10 | Ensure the well-being of health care professionals: there should be a holistic focus on the psychological, physical, and spiritual well-being of health care professionals. Caring for the care team ensures that treatment of IHCA patients remains at a high quality. |
